# Is health financing converging in the European Union? Nonlinear dynamics and policy implications for non-euro area member states

**DOI:** 10.3389/fpubh.2026.1831781

**Published:** 2026-05-20

**Authors:** Melina Dritsaki, Chaido Dritsaki, Panagiotis Sarigiannidis, Alexandra Giola Genni

**Affiliations:** 1 Department of Economics, University of Western Macedonia, Kastoria, Greece; 2 Department of Accounting and Information Systems, International Hellenic University, Thessaloniki, Greece; 3 Department of Electrical and Computer Engineering, University of Western Macedonia, Kozani, Greece; 4 R&D Department, MetaMind Innovations P.C., Kozani, Greece

**Keywords:** convergence, European Union, health expenditure, nonlinear unit root tests, structural breaks

## Abstract

**Background:**

Convergence in health expenditure across European Union (EU) member states is central to health system equity, fiscal sustainability, and regional cohesion. However, whether countries operating outside the euro area are moving toward similar levels of per capita health spending remains unclear, particularly in the presence of economic shocks and institutional heterogeneity.

**Methods:**

This study investigates stochastic convergence in per capita health expenditure among seven EU member states outside the euro area over the period 2000–2022. Using nonlinear and structural-break-sensitive time-series approaches, we assess whether deviations from the group average are temporary or persistent, allowing for asymmetric adjustment and regime shifts.

**Results:**

The findings indicate that health expenditure convergence is neither linear nor uniform across countries. While more fiscally mature and institutionally stable systems exhibit evidence of nonlinear long-run adjustment, several countries display persistent divergence from the group mean. At the panel level, results suggest gradual and cyclical convergence rather than steady alignment. Adjustment dynamics appear sensitive to macroeconomic crises and policy environments, highlighting asymmetric responses during expansionary and contractionary periods.

**Conclusion:**

Health expenditure convergence among EU countries outside the euro area is best understood as a nonlinear and context-dependent process. Persistent divergence in some countries may pose risks for health system equity and resilience, particularly during economic downturns. These findings underscore the importance of adaptive and counter-cyclical health financing strategies at both national and EU levels to promote sustainable convergence and reduce structural disparities in health system capacity.

## 1 Introduction

A key component of human well-being and a crucial sign of socioeconomic advancement is health. Since consistent investment in health promotes productivity, the development of human capital, and long-term economic growth, ensuring that all age groups have fair access to high-quality healthcare services is a fundamental goal of national policy. However, research from both the global and regional levels indicates that health spending is unevenly allocated throughout nations, reflecting differences in institutional strength, fiscal capacity, and income levels. For instance, 80% of the world’s health spending occurs in high-income nations (1), and patterns of per capita spending change over time in response to system-level, social, economic, and demographic factors (2).

From the standpoint of health economics, convergence in per capita health spending indicates the degree to which populations in different nations can anticipate similar access to healthcare resources, service quality, and health outcomes, rather than just budgetary alignment. Particularly during times of financial hardship, persistent difference in health spending runs the danger of solidifying disparities in care delivery, labour capability, and system resilience. Sustained discrepancies in health spending may jeopardise the efficiency and fairness goals of European health systems in the setting of the European Union, where citizen mobility and cross-border healthcare access are legally embedded.

The rising ageing of Europe’s population has coincided with the spread of extremely advanced and expensive medical technologies. The pressure on healthcare spending in all EU member states has increased as a result of these changes. Significant cross-country diversity in terms of financial structures, benefit coverage, and access to primary healthcare persists despite this overall upward trend (3). Additionally, given the flexibility of EU residents to seek healthcare across borders, disparities in quality, adequate finance, and governance capability present additional issues for nations functioning within an integrated regional setting (4). Analysing whether non-EU member states are converging in terms of per capita health spending is both strategically and empirically significant in this context.

Three complementary theoretical stances- economic integration, institutional competence, and health system typologies- can serve as conceptual foundations for the convergence hypothesis. Deeper involvement in EU economic, regulatory, and policy frameworks is anticipated to promote convergence across macro-fiscal variables, including health spending, from the perspective of regional economic integration. Integration facilitates the diffusion of knowledge, harmonisation of standards, greater access to EU structural and cohesion funds and the adoption of common fiscal and regulatory practices. As described in neoclassical growth theory, such conditions support convergence through technology transfer, learning spillovers and reductions in structural inefficiencies.

Institutional capability theory reinforces this argument by emphasising the role of governance, administrative capacity and policy stability in shaping health financing trajectories. Countries with stronger institutions are more likely to translate fiscal resources into effective, stable and predictable long-term health investments, whereas weak institutional environments may lead to underinvestment, inefficiency and volatility, ultimately delaying convergence.

Finally, differences in health system organisational models affect both the scale and evolution of health expenditures. Tax-funded, insurance-based, mixed and market-oriented systems generate distinct expenditure profiles based on coverage design, cost-containment instruments, provider incentives, decentralisation and the public-private mix. Thus, convergence cannot be interpreted purely as an economic catch-up process but must also be understood through system design and governance lenses.

Against this backdrop, the present study examines whether per capita health expenditures among EU member states outside the euro area have converged over the period 2000–2022. Recognising the presence of nonlinear dynamics and context-specific shocks in health expenditure, we employ a suite of nonlinear and structural-break-sensitive unit root tests to assess convergence. By adopting this approach, the study contributes to the limited but growing literature applying nonlinear time-series econometrics to health expenditure patterns, offering deeper insights into whether convergence is linear, nonlinear, symmetric, asymmetric, continuous or cyclical in nature.

This study contributes to the health economics literature in three ways. First, it provides new evidence on health expenditure convergence within a policy- relevant subgroup of EU countries operating outside the euro-area, where fiscal autonomy and health system heterogeneity are particularly pronounced. Second, by employing nonlinear and asymmetric time-series methods, it captures adjustment dynamics that standard linear approaches overlook, offering a more realistic representation of health spending behaviour. Third, the findings inform EU health policy by identifying which health systems exhibit stable convergence and which remain structurally divergent, thereby guiding targeted and efficiency-oriented policy intervention.

The rest of this paper is organized as follows: Section 2 presents literature review. Data and methodology are presented in Section 3. Section 4 describes preliminary analysis, and section 5 presents the unit root tests. The empirical results and discussion are presented in Section 6. Finally, Section 7 presents conclusions and policy implications.

## 2 Literature review

Due in significant part to methodological heterogeneity, variations in nation groupings, time periods, and discrepancies in empirical testing frameworks, research on the convergence of health expenditures has yielded inconsistent and unsatisfactory results. Nonlinear dynamics and structural breaks, which are now acknowledged as crucial components of health spending data, were frequently overlooked in the early research, which mostly relied on linear unit root testing. This discrepancy calls into question the reliability of previous findings and emphasises the need for stronger methodological frameworks that are in line with the data’s stochastic characteristics.

A significant body of empirical research has looked at convergence at the univariate or panel-data level utilising linear or traditional unit root methodologies [e.g., Albulescu et al. (3), Aslan (5), Lau et al. (6), and Narayan (7, 21, 22)]. In most cases, these investigations show little to no evidence of convergence, especially when structural fractures are not taken into account. For instance, Aslan (5) and Lau et al. (6) do not find convergence between OECD and EU nations, respectively. This could be due to methodological omission rather than a real lack of convergence. Similarly, using traditional unit root testing, Albulescu et al. (3) find no mean reversion in health expenditure-to-GDP ratios across a subset of EU member states. Collectively, these findings highlight a potential underestimation of convergence due to linear model assumptions that overlook regime shifts, asymmetries, or nonlinear adjustment paths.

To better capture the intricate dynamics of health spending, more recent research has moved toward nonlinear, asymmetric, and break-inclusive approaches (2, 8–10). When both structural adjustments and nonlinear dynamics are taken into account, this new evidence strengthens the case for convergence, at least for subsets of nations. For example, Pekkurnaz (10) uses nonlinear asymmetric panel methods to find convergence in about 23% of OECD nations, while standard linear methods find none. Similarly, Celik et al. (8) use both linear and nonlinear unit root tests to verify convergence for almost the whole OECD sample. These results highlight the significance of using empirical techniques that may capture state reliance, asymmetric adaptations, and multiple structural breaks, especially when examining lengthy historical series impacted by demographic shocks, economic crises, and policy reforms.

Furthermore, some research has broadened the analysis by examining the ways in which convergence interacts with more general macroeconomic or institutional factors. According to Mutlu et al. (11), convergence in health expenditures may co-evolve with economic convergence, suggesting interdependencies between welfare financing and macroeconomic trajectories. Similarly, (12) study on health expenditure convergence examines the joint convergence of economic growth and health expenditures across OECD countries using both linear and nonlinear unit root tests. Their findings indicate widespread convergence in health expenditure per skilled worker, reinforcing the importance of nonlinear approaches in capturing long-run adjustment dynamics. Singh et al. (13) further distinguish between the convergence of public and private health expenditures, showing differing trajectories across national groupings. This suggests that differences in convergence outcomes may be caused by institutional arrangements, finance regimes, and welfare models.

Despite growing methodological sophistication, several gaps remain unaddressed. First, limited attention has been paid to non-euro-area EU countries, despite their differing macroeconomic frameworks, healthcare financing models, fiscal capacities and structural adjustment pathways. Second, most studies use panel methods, which assume homogeneity and may mask country-specific behaviours; therefore, country-level time-series analyses using nonlinear methodologies remain relatively scarce. Third, many studies incorporate single structural breaks, whereas recent evidence suggests that health expenditure is influenced by multiple cyclical and crisis-driven shocks, including global recessions, evolving medical technologies, and demographic transitions. Fourth, the literature has yet to systematically compare symmetric versus asymmetric convergence, an important consideration given that recessions often produce longer-lasting fiscal effects than expansions especially in healthcare, where demand is price-inelastic. Finally, the role of institutional governance, welfare regimes and policy harmonisation remains insufficiently explored as potential drivers of convergence clubs.

## 3 Data and methodology

### 3.1 Data

This study uses annual data covering the period 2000–2022 for European Union member states that are not part of the euro area. Per capita health expenditure data, expressed in constant 2015 prices and measured in US dollars to ensure temporal comparability and to control for inflationary effects. While Purchasing Power Parity (PPP)-adjusted measures are often preferred for cross-country comparisons of real purchasing power, particularly in sectors dominated by non-tradable services such as healthcare, the primary objective of this study is to examine convergence dynamics rather than absolute expenditure levels. In this context, the use of constant-price series allows for a consistent analysis of time evolution across countries. PPP-adjusted measures, although valuable, may introduce additional variability due to periodic revisions and cross-country methodological differences, which could obscure dynamic patterns, especially within nonlinear econometric frameworks. Nevertheless, PPP-based approaches represent a useful alternative and are highlighted as a potential avenue for future research. In this study, per capita health expenditure data are obtained from the OECD Health Expenditure and Financing Database (2023). Health expenditure per capita is widely used in the health economics literature as a summary indicator of health system financing intensity and cross-country comparability.

**Figure 1:**
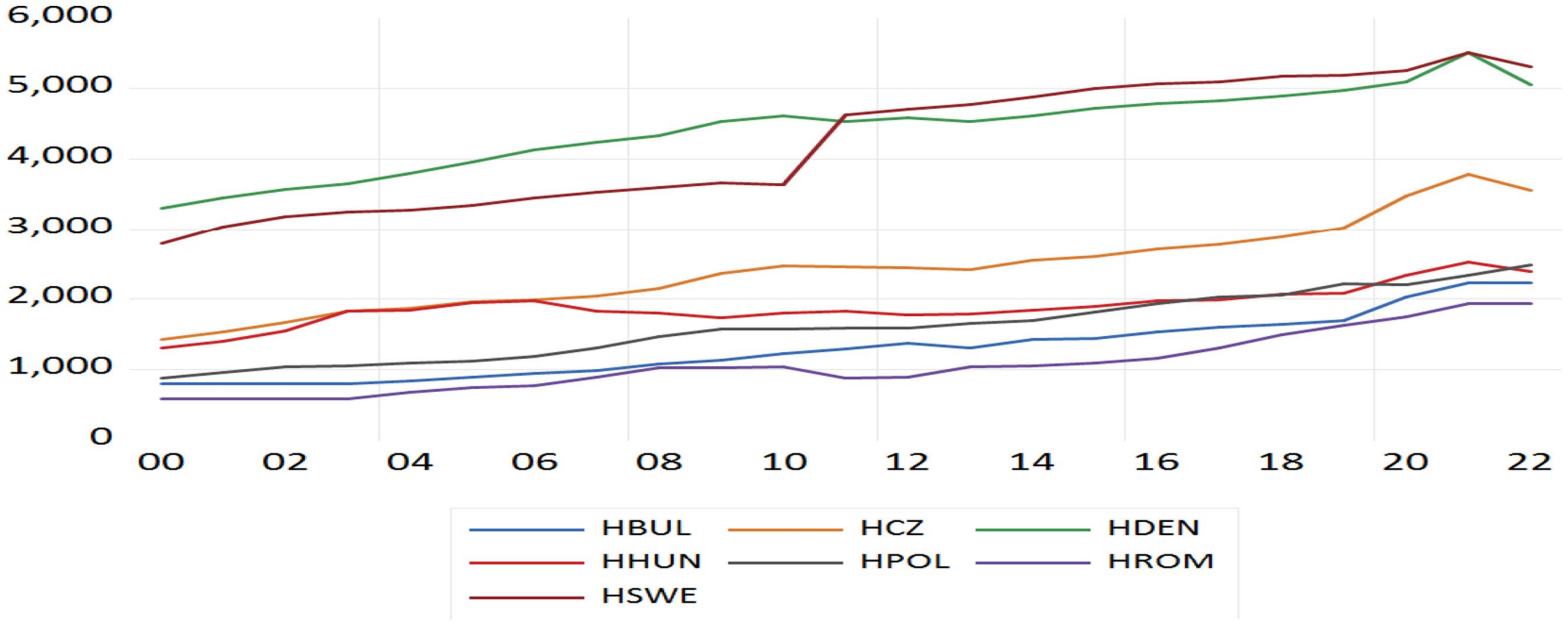
Time series of health expenditure per capita from 2000 to 2022.

Figure 1 below shows the evolution of per capita health expenditures in EU countries outside the Eurozone during the period 2000–2022.

Based on the figure above, a long-term upward trend in per capita health expenditures is observed across all countries included in the analysis. The data further reveal the presence of a structural change in 2021, characterised by a decline in per capita health spending in all EU member states outside the euro area, as well as additional country-specific changes in particular sub-periods. Specifically, an increase in expenditures is recorded in Sweden during 2010–2011, in the Czech Republic, Hungary and Bulgaria during 2019–2021, and in Denmark during 2020–2021. Notably, the downturn observed in 2021 appears to be widespread across the sample. This decline may be associated with the fiscal consequences of the Covid-19 pandemic, during which several governments temporarily constrained non-essential public health expenditures and private out-of-pocket spending decreased due to reduced economic activity. The 2021 decline therefore represents a potential structural break, consistent with the results of unit root tests that allow for regime shifts, which also detected similar breakpoints for certain countries.

In addition, the substantial increase in health expenditures observed in Sweden during 2010–2011 may reflect policy-driven reinforcement of the healthcare sector in the aftermath of the global financial crisis. Likewise, the upward trend in the Czech Republic, Hungary and Bulgaria between 2019–2021 may be linked to increased public investment in health systems and strategic efforts to align with European health-financing standards. Denmark’s increase during 2020–2021 may similarly indicate enhanced public budget allocations aimed at pandemic management and the maintenance of service quality.

Overall, the graphical evidence suggests that, despite the common downward shift in 2021, countries follow distinct long-term expenditure trajectories, reinforcing the existence of heterogeneity in spending dynamics. This pattern implies that convergence in per capita health expenditures among EU member states outside the euro area remains incomplete and gradual rather than uniform.

Table 1, presents the descriptive statistics for all the countries under examination.

**Table 1:** Descriptive statistics.

Country	Mean	Standard deviation	Skew.	Kurt.	J.Β	Prob.	Obs.
Bulgaria	1308.17	448.57	0.663	2.547	1.885	0.389	23
Czechia	2433.26	630.37	0.456	2.611	0.945	0.623	23
Denmark	4420.73	579.72	−0.358	2.352	0.893	0.639	23
Hungary	1890.56	280.12	0.227	3.478	0.417	0.811	23
Poland	1602.78	475.25	0.178	1.937	1.203	0.547	23
Romania	1072.21	424.09	0.772	2.590	2.449	0.293	23
Sweden	4232.04	910.86	−0.092	1.343	2.661	0.264	23
HEE	2422.82	1389.8	0.736	2.317	17.67	0.000	161

Source: Author’s calculations.

Analysis of the descriptive statistics reveals substantial variation among EU member states outside the euro area with respect to per capita health expenditures. Denmark records the highest average per capita spending, reaching 4,420.73 USD, accompanied by relatively low dispersion (standard deviation: 579.72), indicating a consistently high and stable level of expenditure over time. In contrast, Romania exhibits the lowest spending, with an average of 1,072.21 USD and a standard deviation of 424.09, reflecting both limited public and private health financing and potential structural weaknesses within the national healthcare system.

The overall mean per capita health expenditure across all countries is 2,422.82 USD, with a high standard deviation of 1,389.8, signalling considerable heterogeneity and pronounced inequalities in expenditure levels across countries. These findings reflect cross-country differences in stages of economic development, fiscal capacity, and health system policy priorities. Collectively, the descriptive results suggest that EU countries outside the euro area do not constitute a homogeneous group in terms of health spending patterns, thereby justifying further econometric investigation into their convergence dynamics and underlying determinants.

Socio-economic convergence is typically examined within the context of two dominant economic growth theories: the neoclassical and endogenous growth frameworks. According to traditional growth theory, three core forms of convergence may be assessed. β-convergence tests whether countries with lower initial health expenditure levels experience faster growth than high-expenditure countries; this is usually estimated through regressions of expenditure growth on initial levels. σ-convergence evaluates whether the cross-country dispersion (e.g., standard deviation or variance) of the variable declines over time. Stochastic convergence tests whether deviations of individual countries from a group benchmark are stationary and not persistently diverging, typically assessed through linear and nonlinear unit root tests.

In the present study, we examine the convergence of per capita health expenditures among EU member states outside the euro area using a stochastic convergence approach. To examine stochastic convergence, per capita health expenditure for each country is transformed into a relative measure by taking the natural logarithm of the ratio of national expenditure to the cross-country average. Specifically, the transformed series is defined as the log deviation of country-specific health expenditure from the group mean. This transformation allows the analysis to focus on whether deviations from the common benchmark are temporary (stationary) or persistent (non-stationary), which constitutes the standard approach in stochastic convergence analysis. Specifically, health expenditure per capita for country i relative to the group mean at time t is constructed as follows in equation 1 below:


$$y_{\mathrm{it}}=\ln(\frac{h_{\mathrm{it}}}{{\bar{h}}_{t}})$$ (1)

Where $h_{\mathrm{it}}$ it is per capita health expenditure for country *i* at time *t*, ${\bar{h}}_{t}$ is the average per capita health expenditure at time t and ln is the natural logarithm.

**Figure 2:**
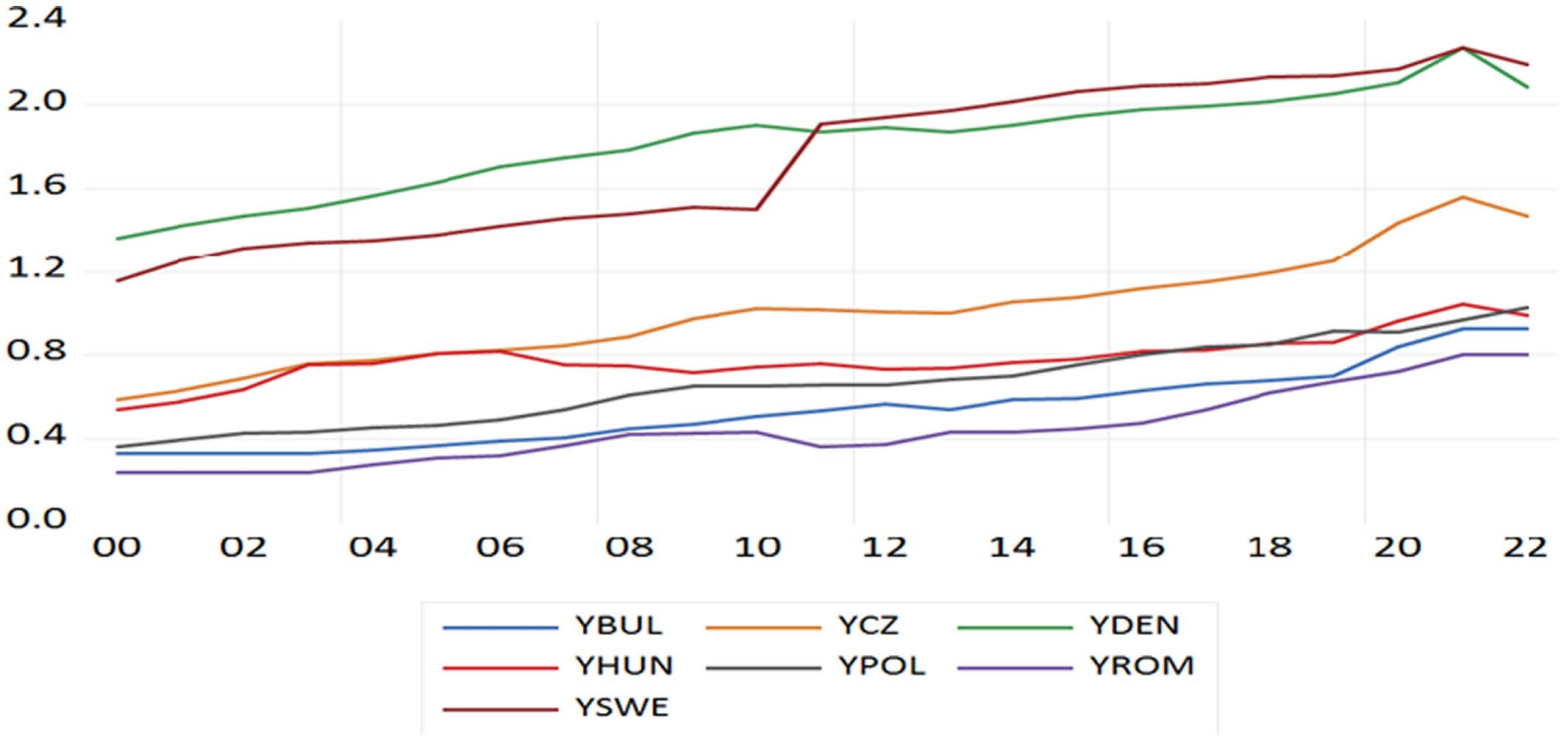
Health expenditure per capita of country relative to average per capita health expenditures among in EU countries outside the Eurozone.

Figure 2 shows the evolution of per capita health expenditure in EU countries outside the Eurozone over the period 2000–2022 in relation to the average per capita health expenditure among the 7 EU countries.

A limitation of the present study relates to the relatively short time dimension (*T* = 23), which is inherent to the use of annual data over the period 2000–2022. Although this sample size is consistent with a large body of empirical macroeconomic literature and reflects data availability constraints for comparable cross-country health expenditure series, it may reduce the statistical power of econometric tests, particularly those based on asymptotic properties. While the nonlinear unit root approaches employed in this study, such as Omay et al. (14), are designed to perform adequately in small samples, the results should nevertheless be interpreted with appropriate caution.

The selection of countries is based on European Union member states that are not part of the euro area, in order to examine convergence dynamics within a group characterised by greater fiscal autonomy and institutional heterogeneity. The study period (2000–2022) is determined by the availability of consistent and comparable data on per capita health expenditure across all selected countries. Earlier periods were excluded due to data limitations and inconsistencies in reporting methodologies.

### 3.2 Why nonlinear methods are required in health expenditure analysis

Health expenditure dynamics are shaped by institutional reforms, fiscal constraints, demographic change and exogenous shocks such as financial crises and public health emergencies. As a result, adjustment paths are unlikely to be smooth, symmetric or time-invariant. In practice, health spending often responds differently to economic expansions than to downturns, with reductions during periods of fiscal stress frequently being more abrupt or persistent than increases during periods of growth.

Standard linear unit root tests impose restrictive assumptions of symmetric and stable adjustment, which may be inappropriate for health expenditure data. Failure to account for nonlinearity and structural change can therefore lead to misleading conclusions regarding convergence. In the context of European health systems, particularly among countries outside the euro area, where fiscal autonomy and institutional heterogeneity are pronounced, methods that allow for asymmetric behaviour and regime shifts are especially relevant.

For these reasons, this study adopts a set of nonlinear, asymmetric and structural-break-sensitive unit root tests that are capable of capturing heterogeneous adjustment dynamics and multiple structural changes over time.

### 3.3 Empirical strategy and overview of methods

The empirical framework follows a univariate time-series approach to stochastic convergence, where the focus is on the dynamic behaviour of deviations from a common benchmark rather than on multivariate determinants of expenditure levels. As a result, no additional control variables are included in the model specification (1). This approach is consistent with the convergence literature, where unit root testing is applied to transformed series to assess whether shocks to relative expenditure are temporary or permanent.

Based on the empirical framework, the study tests the following hypotheses:

H0: Per capita health expenditure does not converge across countries (the transformed series contains a unit root).

H1: Per capita health expenditure converges across countries (the transformed series is stationary, potentially in a nonlinear and/or asymmetric manner).

Given the presence of structural breaks and nonlinear dynamics in health expenditure data, it is expected that linear tests may fail to detect convergence, while nonlinear and structural-break-sensitive tests may provide stronger evidence of convergence.

The empirical analysis proceeds in three steps. First, the presence of nonlinear dependence in the health expenditure series is assessed. Second, convergence is examined using unit root tests that allow for structural breaks and nonlinear adjustment. Third, the robustness of convergence dynamics is evaluated by employing alternative specifications that capture asymmetric behaviour and multiple smooth structural changes.

The transformed health expenditure series is subjected to the Brock–Dechert–Scheinkman (15) test to determine whether the data-generating process has nonlinear dependence. The use of nonlinear unit root tests in further investigation is justified by evidence of nonlinearity.

A structural-break-sensitive unit root framework is then used to analyse convergence. To take into consideration the potential for a single endogenously determined structural break in health expenditure trajectories, the Zivot–Andrews (16) test is utilised. This strategy is especially important when there are significant policy changes or macroeconomic upheavals that could change long-term expenditure trends.

A series of unit root tests based on exponential smooth transition autoregressive (ESTAR) are used to capture nonlinear mean reversion. While the Kruse (17) test loosens the requirement of a zero location parameter, the Kapetanios–Shin–Snell-KSS (18) test looks at symmetric nonlinear adjustment. Furthermore, the Sollis (19) test permits asymmetric adjustment, acknowledging that the convergence trajectories of upward and downward variances in health spending may differ. Since responses to budgetary expansions and contractions are unlikely to be symmetric, these approaches work well with data on health spending.

Lastly, the Omay et al. (14) paradigm is used to explain various smooth structural changes and regime-dependent behaviour. This method allows the analysis to capture both asymmetric adjustment around nonlinear trends and gradual structural alterations by combining nonlinear unit root testing with logistic and Fourier trend definitions. When studying lengthy health spending series impacted by recurring reforms, demographic shifts, and cyclical macroeconomic situations, this flexibility is crucial.

When taken as a whole, this multi-layered approach provides a more realistic picture of the dynamics of health system adjustment among EU member states outside the euro area by enabling a thorough evaluation of health spending convergence that goes beyond linear and time-invariant assumptions.

All tests are conducted at the country level, allowing for heterogeneous convergence behaviour across health systems. Lag lengths are selected using information criteria, and critical values are drawn from the relevant test-specific distributions (20). Full technical specifications, test equations and implementation details are provided in the Supplementary Appendix.

## 4 Empirical results

### 4.1 Linear and non-linear tests (BDS independence test)

The following Table presents the results of the Brock et al. (15) test for the independence of the variable. The Brock et al. (15) test detects the nonlinearity of the variable as well as the serial dependence of the time series data. The null hypothesis assumes that the series is linearly independent and identically distributed (i.i.d.). The alternative hypothesis assumes that the series is nonlinear or that the series exhibits serial dependence. *p*-values were estimated with bootstrap (1,000 replicates).

The results of the Table 2 show rejection of the null hypothesis (significance level less than 1%). Therefore, we can say that there is nonlinearity in the variable we are examining or serial dependence in all countries. The test is conducted in increasing dimensions (from Dim 2 to Dim 6), with higher dimensions capturing more complex dependencies. As the BDS statistic increases in higher dimensions, this indicates stronger evidence of nonlinearity, or serial dependence, in the variable. Rejection of the null hypothesis in all dimensions confirms the presence of nonlinearity and serial dependence in the data for all countries. The nonlinearity of per capita health spending means that changes in per capita health spending in EU countries outside the eurozone do not increase or decrease at a constant rate but vary depending on the level or stage of development of each country. Furthermore, expenditures do not evolve steadily, i.e., there are periods of rapid growth (after reforms, income growth, or fiscal support) and periods of slowdown or reduction (austerity, crisis, budget constraints). Also, the relationships between income, public spending and health spending are likely to be asymmetric, that is, an increase in GDP may lead to a larger increase in spending, but a decrease in GDP may lead to a much larger or smaller decrease. There are structural differences between countries, some with developed health systems (Denmark, Sweden) present stable and smooth expenditures, while others (Romania, Bulgaria) exhibit instability and non-linear fluctuations.

**Table 2:** BDS independence test.

BDS statistic	Dim 2	Dim 3	Dim 4	Dim 5	Dim 6
Bulgaria	0.138 (0.00)	0.183 (0.00)	0.171 (0.00)	0.170 (0.00)	0.106 (0.00)
Czechia	0.148 (0.00)	0.196 (0.00)	0.191 (0.00)	0.199 (0.00)	0.212 (0.00)
Denmark	0.185 (0.00)	0.289 (0.00)	0.389 (0.00)	0.464 (0.00)	0.519 (0.00)
Hungary	0.127 (0.00)	0.140 (0.00)	0.121 (0.00)	0.185 (0.00)	0.228 (0.00)
Poland	0.164 (0.00)	0.255 (0.00)	0.322 (0.00)	0.355 (0.00)	0.357 (0.00)
Romania	0.129 (0.00)	0.155 (0.00)	0.097 (0.00)	0.022 (0.00)	0.119 (0.00)
Sweden	0.189 (0.000)	0.309 (0.000)	0.397 (0.000)	0.458 (0.000)	0.487 (0.00)

Source: Author’s calculations.

Non-linearity in per capita health spending in non-eurozone countries may be due to:

- Fiscal policy. Alternating austerity policies and spending expansion create discontinuities in the flow of funds.
- Income changes. Health spending increases more when income increases but does not decrease as much when income falls.
- Institutional reforms. Reforms in insurance or public health systems cause sharp jumps in spending.
- Exogenous shocks. Pandemics, economic crises or fiscal changes cause temporary deviations from the linear trend

Non-linearity in per capita health spending may reflect:

- Different degrees of prosperity and fiscal stability.
- Heterogeneity in national health systems.
- Different monetary and exchange rate policies that affect the ability to finance the health sector.
- Reaction to crises (COVID-19, inflation, energy crisis) in a non-consistent manner depending on the country and the year.

The existence of nonlinearity in per capita health spending suggests that the evolution of spending does not follow a stable or predictable path, but varies depending on economic and institutional factors. In EU countries that are not part of the euro area, the nonlinear behaviour can be attributed to income fluctuations in fiscal austerity policies or expansions as well as to structural changes in health systems. This fact suggests that changes in health spending are not symmetrically affected by changes in economic conditions, which justifies the use of nonlinear models to accurately capture the relationship between macroeconomic variables and health spending.

Since there is nonlinearity in per capita health expenditures, we can examine their stationarity with structural change tests, as well as nonlinear unit root tests.

### 4.2 Unit root testing

#### 4.2.1 Zivot-Andrews test

The results of Zivot and Andrews (16) test of per capita health expenditure are presented in Table 3.

**Table 3:** Unit root test with structural breaks results.

Zivot-Andrews									
Level									
	C			T			C, T		
	k	t-statistic	break-point	k	t-statistic	break-point	k	t-statistic	break-point
Bulgaria	0	−2.788	2019	0	−4.247***	2019	0	−4.059	2018
Czechia	1	−5.028**	2019	1	−5.025*	2018	1	−6.117*	2015
Denmark	0	−4.69***	2005	0	−4.013	2009	0	−4.940***	2011
Hungary	0	−3.074	2007	0	−2.934	2016	0	−2.708	2007
Poland	0	−2.553	2011	0	−2.348	2015	0	−2.851	2012
Romania	1	−3.055	2011	1	−3.310	2016	1	−4.442	2011
Sweden	0	−13.934*	2011	0	−2.529	2017	0	−13.178*	2011

Critical values. Intercept: −5.34 (1%), −4.93 (5%), −4.58 (10%); trend: −4.80 (1%), −4.42 (5%), −4.11 (10%); both: −5.57 (1%), − 5.08 (5%), −4.82 (10%).

Note: The test includes the intercept term. k is the optimal lag length suggested by the Akaike information criterion (AIC). The maximum lag length is 4. *, **, and *** indicate significance at the 1, 5 and 10% level, respectively. C, constant; T, trend.

Source: Author’s calculations.

The results of the above table show that per capita health expenditure presents a unit root in all three Zivot and Andrews (16) control functions in Hungary, Poland, and Romania. In contrast, Czechia rejects the unit root in all three functions with structural changes in the years 2019, 2018, and 2015, respectively. Bulgaria rejects the unit root in the Zivot-Andrews function with the trend function and with structural change in 2019. Denmark rejects the unit root in the Zivot-Andrews function with the constant and constant and trend function and with structural change in 2005 and 2011, respectively. Sweden rejects the unit root in the Zivot-Andrews function with the constant and the constant and trend function with structural change in the year 2011 in both functions and at a significance level of 1%.

The results of the Zivot and Andrews (16) test for per capita health expenditure in non-euro area EU countries show differentiated dynamics across countries, which is of direct relevance for investigating health expenditure convergence. Specifically, Hungary, Poland, and Romania exhibit a unit root in all three functions. This finding suggests that per capita health spending in these countries is non-stationary, meaning it does not return to a long-term equilibrium level. In other words, we would say that spending follows an increasing path over time without stabilizing or converging toward the level of other countries. This behaviour may reflect the ongoing process of modernization of health systems and the gradual adaptation to European standards, but without a stable convergence with the most developed countries having yet been achieved. In contrast, the Czech Republic rejects the null hypothesis in all three functions, showing stagnation with structural changes in 2015, 2018 and 2019. This result shows that Czech health spending is returning to a long-term equilibrium level after specific structural changes, which is linked to the convergence toward spending levels in more developed countries. Similarly, Bulgaria shows stagnation only in the trend function with structural change in 2019, indicating that the country is following a steady upward trend in health spending that is gradually adjusting, but at a slower pace, toward European levels. Regarding the Northern European countries, Denmark and Sweden reject the unit root in most control specifications with structural changes in 2011. This suggests that health spending in these countries is stagnant around a constant level, despite temporary fluctuations caused by external events (such as the economic crisis or fiscal adjustments). These countries are therefore already in a state of convergence and stability of health spending.

Overall, the results indicate that:

- The most developed countries (Denmark, Sweden, Czech Republic) are experiencing stagnation with structural changes, which means that they have achieved convergence to stable levels of health spending.
- Eastern European countries (Hungary, Poland, Romania) exhibit non-stationary behaviour, which indicates a lack of convergence and an ongoing adjustment process.
- Bulgaria appears to be in an intermediate stage with partial stagnation and recent changes that may signal the beginning of a convergence path.

The results of the Zivot-Andrews audit demonstrate that the convergence of per capita health spending in EU countries outside the euro area is not complete, but is gradually evolving. Northern and Central European countries have already stabilized around a long-term spending level, while Eastern European countries are still on an adjustment path.

#### 4.2.2 Nonlinear unit root tests

Table 4 presents the results of the nonlinear tests of Kapetanios et al. (18) and Kruse (17) of a symmetric ESTAR model and Sollis (19) of an asymmetric ESTAR model (AESTAR).

**Table 4:** Nonlinear unit root tests results.

Country	KSS( $t_{\mathrm{NL}}$ )		Kruse( τ )		Sollis		
	*k*	Stat	*k*	Stat	*k*	$H_{0}:\phi_{1}=\phi_{2}=0$	$H_{0}:\phi_{2}=0$
Bulgaria	0	3.555	0	9.876	0	11.043*	3.248
Czechia	0	2.532	0	6.878	1	4.843***	2.943
Denmark	1	2.031	0	6.973	0	8.147*	3.729
Hungary	0	1.421	0	2.578	0	2.311	1.742
Poland	0	4.849	0	17.919*	0	15.593*	2.707
Romania	0	2.917	0	7.665	0	8.566*	3.030
Sweden	0	1.356	0	4.252	0	4.435***	2.649
E.E	1	3.461	0	36.445*	0	32.858*	7.499*

The symbols ^*^, ^**^, and ^***^ mean rejection of the null hypothesis of unit root at the 1, 5 and 10%, respectively.

KSS: −3.48, −2.93, −2.66; Kruse: 13.75, 10.17, 8.6; Sollis: 6.883, 4.954, 4.157.

The results of the above table show that the KSS test does not reject the null hypothesis in any of the countries we examine. That is, according to the KSS test, per capita health expenditure is non-stationary. Therefore, we can say that there is no convergence in a linear framework. The results also show that the Kruse test rejects the null hypothesis only for Poland as in the panel data. This suggests that only Poland exhibits nonlinear stationarity, i.e., partial convergence. The Sollis test shows stationarity in all non-euro area EU countries (except Hungary) as in the panel as a whole. This means that per capita health expenditure is non-linearly stationary, meaning that countries tend to converge in the long run, but not symmetrically. However, the asymmetric stationarity is only found in the panels, not in each country separately, so the asymmetry of the adjustment seems to be a collective (pan-European) phenomenon and not an isolated one.

These results show that the convergence of per capita health spending in EU countries outside the eurozone is not linear, but exhibits nonlinear and partly asymmetric behaviour. That is, differences between countries in per capita health spending do not decrease in a steady and linear manner, but are followed by periods of rapid convergence when the differences are large (after crises or reforms) and slow adjustment when the differences are small.

The presence of nonlinear and asymmetric adjustment has important implications for health system performance. It indicates that periods of fiscal expansion or reform may enable rapid improvements in health spending and service capacity, whereas downturns and austerity episodes may generate prolonged setbacks. Such asymmetry implies that health systems in lower-spending countries are more vulnerable to economic shocks, potentially widening inequalities in access to care and health outcomes during adverse periods.

The stationarity that Sollis identifies suggests that, in the long run, expenditures tend to adjust toward a common equilibrium level, but this process is nonlinear, that is, the speed of adjustment depends on the magnitude or direction of the deviation.

The results of the nonlinear unit root tests demonstrate that the convergence process of per capita health expenditure in EU countries outside the eurozone is not linear. The KSS test does not confirm stationarity, which rejects the existence of linear convergence. However, the Kruse and Sollis test shows nonlinear stagnation for some countries such as Poland and for the entire panel, suggesting that health spending adjusts toward a common level in a nonlinear and asymmetric manner. Therefore, it can be argued that there is non-linear long-term convergence of per capita health spending, which manifests itself mainly at the collective level and not individually in each country.

Table 5 presents the results of the two specifications of Omay et al. (14), namely logistic trend function and Fourier trend function. The F-statistic is referred to as FLBAE for the logistic smooth transition (LTR) of models A, B and C, and as FFSAE for the Fourier functions. Omay et al. (14) obtain the critical values of FLBAE and FFSAE through stochastic simulation and show that the tests are valid even in small sample sizes.

**Table 5:** Omay et al. (14) unit root test results.

Country	Logistic trend function specification (F_LBAE_)			Fourier trend function specification (F_FSAE_)	
	Model A	Model B	Model C	C	C + T
Bulgaria	4.752	4.981	6.703	3.763 (1)	4.873 (1)
Czechia	8.377**	8.465***	11.532**	5.032 (1)	6.123 (1)
Denmark	7.231***	5.891	9.421***	4.976 (1)	6.365 (1)
Hungary	2.943	3.212	4.426	3.067 (1)	4.126 (1)
Poland	5.432	3.873	6.733	4.783 (1)	4.956 (1)
Romania	3.876	6.421	6.642	2.977 (1)	4.652 (1)
Sweden	11.067*	7.745	12.734**	6.92 (1)**	8.14 (1)**
E.E	16.523*	9.114***	10.817**	17.44 (3)*	28.482 (4)*
Test critical values					
1%	10.756	12.681	13.621	8.68	10.61
5%	8.110	9.642	10.617	6.36	7.93
10%	7.101	8.339	9.209	5.31	6.75

*, **, and *** indicate significance at the 1, 5 and 10% level, respectively. C, constant; T, trend. The numbers in parenthesis in F_FSAE_ indicate the number of frequency components in the Fourier series.

Source: Author’s calculations.

The results of the test by Omay, Emirmahmutoglu, and Hasanov for the first specification (logistic trend function) show that the null hypothesis cannot be rejected for the countries Bulgaria, Hungary, Poland, and Romania in all three test models. The failure to reject the null hypothesis for Bulgaria, Hungary, Poland, and Romania means that these countries do not exhibit stagnation, that is, they do not exhibit convergence of per capita health spending toward a common level. Possible explanations include structural differences in health systems, lower levels of economic development, and limited public spending.

On the contrary, the null hypothesis is rejected for the countries Czechia, Denmark, and Sweden, as well as in the panel data for the first model of the logistic trend function. In the second model, the null hypothesis is rejected only for Czechia and in the panel data, while in the third model of the logistic trend function the null hypothesis is rejected for the countries Czechia, Denmark, and Sweden, as well as in the panel data. Rejection of the null hypothesis for the countries Czechia, Denmark, and Sweden in the first and third models and in the panel data means that these countries exhibit nonlinear stagnation around a nonlinear trend with asymmetric convergence of magnitude nonlinearity, so there is long-run convergence of health spending. Convergence appears to be faster in these countries, where health systems are more developed and public policies are stable. The fact that the panel data reject the null hypothesis in all three models suggests that there is an overall trend of nonlinear convergence at the group level, even if not across all countries.

The second specification (Fourier trend function) of the Omay, Emirmahmutoglu and Hasanov test shows that the null hypothesis is rejected only in Sweden as well as in the panel data with both constant and constant and trend. Rejection of the null hypothesis for Sweden only and the panel data implies that the series is stationary around multiple structural changes in trend with asymmetric convergence of magnitude nonlinearity. Furthermore, this suggests that only Sweden exhibits cyclical nonlinear stagnation, i.e., adjustment with a wavy periodic behaviour. At the panel level, the existence of stagnation indicates a collective convergence trend where countries adjust in the long run toward a common equilibrium point, but in a nonlinear and cyclical manner.

The results of the Omay, Emirmahmutoglu and Hasanov audit demonstrate that the convergence process of per capita health spending in non-euro EU countries is not linear but asymmetric and non-linear. The first specification (logistic trend function) shows that some countries such as the Czech Republic, Denmark and Sweden exhibit stagnation around a structural change in trend, which suggests nonlinear asymmetric convergence. The second specification (Fourier trend function) reveals cyclical adjustment with multiple structural changes both in Sweden and in the entire panel, which demonstrates that, although there are differences between countries, all EU countries outside the eurozone show long-term nonlinear and cyclical convergence of per capita health spending.

Cyclical and regime-dependent convergence patterns suggest that health expenditure alignment across non-euro EU countries is highly sensitive to macroeconomic and policy environments. For health systems, this implies that convergence achieved during favourable periods may not be durable unless supported by institutional resilience and counter-cyclical financing mechanisms. Without such safeguards, temporary convergence may reverse during crises, with long-term consequences for equity and continuity of care.

## 5 Discussion

The findings of this study provide new insights into the convergence dynamics of per capita health expenditure among European Union member states outside the euro area. The results consistently indicate that convergence is not linear, but rather exhibits nonlinear, asymmetric and regime-dependent characteristics. This finding aligns with a growing body of literature suggesting that conventional linear approaches may underestimate convergence processes when structural breaks and nonlinear adjustment mechanisms are present.

In contrast to earlier studies relying on linear unit root tests [e.g., Albulescu (3), Aslan (5), and Lau et al. (6)], which often report weak or no evidence of convergence, our results support the view that convergence may exist but follows a nonlinear path. This is consistent with more recent contributions such as (12) study on health expenditure convergence, who demonstrate that nonlinear methods reveal convergence patterns that remain undetected under linear specifications. The present study extends this line of research by focusing specifically on non-euro-area EU countries, a group characterised by greater fiscal and institutional heterogeneity.

A key contribution of the analysis lies in highlighting differences in convergence behaviour across country groups. While countries with more developed and stable health systems, such as Denmark and Sweden, exhibit evidence of stationarity and convergence around long-run equilibria, others including Hungary, Poland and Romania, display persistent divergence. This heterogeneity suggests that convergence is conditional on institutional capacity, fiscal stability and health system maturity. Compared to studies focusing on euro-area countries, where stronger fiscal coordination and policy alignment may promote more uniform convergence patterns, the present findings indicate that countries outside the euro area experience more fragmented and uneven adjustment dynamics.

The identification of asymmetric and nonlinear adjustment mechanisms also has important implications. It suggests that health expenditure convergence is highly sensitive to macroeconomic shocks and policy environments, with periods of crisis potentially slowing or reversing convergence processes. This is consistent with the broader literature on nonlinear macroeconomic adjustment, where downturns often have more persistent effects than expansions. In the context of health systems, such asymmetries may translate into widening disparities in access to care and system capacity during periods of fiscal stress.

Overall, the results reinforce the importance of adopting flexible and context-sensitive analytical frameworks when examining health expenditure dynamics. By incorporating nonlinearities and structural breaks, the present study provides a more realistic representation of convergence processes and contributes to a more nuanced understanding of health system evolution across European countries.

## 6 Conclusion and policy implications

This study examined whether per capita health expenditures among EU member states outside the euro area have converged over the period 2000–2022, employing a suite of nonlinear, asymmetric and structural-break-sensitive time-series methods. The empirical evidence indicates that health expenditure convergence in this group of countries cannot be characterised as linear or uniform. Instead, adjustment dynamics are heterogeneous, nonlinear and, in several cases, asymmetric, with convergence occurring only partially and primarily among more fiscally mature and institutionally stable health systems. At the panel level, the results suggest a gradual and cyclical form of long-run convergence rather than steady alignment, highlighting the importance of accounting for regime shifts and nonlinear behaviour when assessing health expenditure dynamics.

The results also show that a number of nations, especially Hungary, Poland, and Romania, have non-stationary spending patterns, which show limited automatic catch-up over time and ongoing divergence from the group average. Denmark, Sweden, and the Czech Republic, on the other hand, show signs of stationarity around structurally changing trends, which is consistent with health systems that have attained comparatively stable long-term finance trajectories. When considered collectively, these findings indicate that the harmonisation of health spending among EU member states that are not part of the euro area is still not complete and is susceptible to institutional features, budgetary adjustments, and macroeconomic situations.

This study contributes to the existing literature in several important ways. First, it provides new empirical evidence on health expenditure convergence within a relatively underexplored group of EU countries operating outside the euro area, where institutional and fiscal heterogeneity is particularly pronounced. Second, by employing nonlinear, asymmetric and structural-break-sensitive econometric techniques, it captures adjustment dynamics that conventional linear approaches may overlook, thereby offering a more nuanced understanding of convergence processes. Third, the findings extend the policy relevance of convergence analysis by linking expenditure dynamics to issues of health system equity, resilience and fiscal sustainability.

Notwithstanding these contributions, several limitations should be acknowledged. First, the relatively short time dimension of the dataset (*T* = 23) may constrain the statistical power of the applied econometric methods, particularly those relying on asymptotic properties. Although the selected techniques are suitable for small samples, future research using longer time spans or higher-frequency data could further strengthen the robustness of the results. Second, the use of per capita health expenditure in constant US dollars, while appropriate for analyzing temporal dynamics, may not fully capture cross-country differences in purchasing power. Third, the analysis focuses primarily on expenditure dynamics and does not directly incorporate health outcomes or institutional quality indicators, which may also play a critical role in shaping convergence processes.

Future research could build on the present analysis in several directions. First, extending the time horizon or employing higher-frequency data, where available, would allow for more robust inference and improved identification of long-run dynamics. Second, incorporating Purchasing Power Parity (PPP)-adjusted measures of health expenditure could provide complementary insights into convergence in real resource allocation. Third, future studies could integrate health outcomes, institutional quality, and demographic variables to better capture the multidimensional nature of health system convergence. Finally, the application of panel nonlinear techniques or convergence club methodologies may further enhance understanding of heterogeneous adjustment patterns across countries.

These results have significant ramifications for health economics research and European health policy in addition to their econometric relevance. They emphasise, in particular, that convergence in health spending should be viewed as a factor that determines the equality, resilience, and long-term effectiveness of the health system as well as a fiscal or macroeconomic process.

### 6.1 Health system equity

Periods of economic crisis may disproportionately increase health spending disparities across EU member states outside the euro area, according to evidence of nonlinear and asymmetric convergence. Such difference runs the potential of resulting in long-lasting disparities in healthcare outcomes, service quality, and access in the absence of remedial EU-level processes. The ability of nations with non-stationary spending pathways to absorb shocks and maintain fair care delivery may be hampered by sustained underinvestment in preventative services, labour capacity, and health infrastructure. These results emphasise how crucial it is to keep an eye on convergence as a budgetary trend and as a factor influencing equity throughout European health systems.

### 6.2 Health financing governance

Uniform health financing targets might not be enough to support long-term convergence across diverse health systems, according to the reported asymmetric and regime-dependent adjustment processes. Rather, the results encourage the creation of EU health financing tools that are flexible rather than consistent, acknowledging that nations react differently to institutional changes, economic shocks, and demographic pressures. By stabilising health spending during recessions and promoting efficiency-focused investment during times of expansion, flexible and countercyclical finance regimes may improve system resilience. This strategy is in line with the larger EU goals of long-term health system performance and fiscal sustainability.

Overall, our study shows that rather than a deterministic trajectory, health expenditure convergence among EU nations outside the euro area is better characterised as a nonlinear and context-dependent process. The analysis offers a more complex understanding of convergence processes and their consequences for equality and governance in European health systems by emphasising the importance of asymmetries, structural changes, and institutional variability. In order to better inform evidence-based policy formulation at the national and EU levels, future research might expand this paradigm by directly tying expenditure convergence to health outcomes and system efficiency.

## Data Availability

The original contributions presented in the study are included in the article/Supplementary material, further inquiries can be directed to the corresponding author/s.
