# Supplementary material for: Is health financing converging in the European Union? Nonlinear dynamics and policy implications for non-euro area member states
Source: Front Public Health. 2026 May 20;14:1831781. doi: 10.3389/fpubh.2026.1831781 (PMC13231585; doi:10.3389/fpubh.2026.1831781)
Supplement: Supplementary file 1 [file Supplementary_file_1.DOCX]

Supplementary Material

**Preliminary Analysis**

**1.Linear and non-linear tests (BDS independence test)**

To examine whether the series follows a linear or nonlinear data-generating process, we apply the Brock, Dechert and Scheinkman (BDS, 1987) statistic. The BDS test serves as a diagnostic tool for detecting nonlinear dependence and serial correlation within the series. The null hypothesis assumes that the series is independent and identically distributed (i.i.d.), which implies linearity and the absence of serial dependence, while the alternative hypothesis suggests the presence of either nonlinear structure or serial dependence (Dritsaki & Dritsaki, 2025).

The BDS test is grounded in the concept of spatial correlation derived from chaos theory and relies on the correlation dimension to detect nonlinear dynamics in a time series. Furthermore, it can be employed to evaluate the adequacy of model specification by assessing whether residuals behave as i.i.d. noise. The BDS testing procedure is implemented following the methodological steps outlined by Chu (2001).

- Let be the time series$\left\{x_{i}\right\}=(x_{1},x_{2},...x_{N})$ with N observations, which should be the first difference of the natural logarithms of the data in the series.
- We choose a value$m$ as the embedding dimension and embed it in the time series in $m$-dimensional vectors, taking each $m$ successive point in the series. This converts the series of scalars into a series of vectors with overlapping entries.

$$\begin{matrix} x_{1}^{m}=(x_{1},x_{2},...,x_{m}) \\ x_{2}^{m}=(x_{2},x_{3},...,x_{m+1}) \\ ... \\ ... \\ ... \\ x_{N-m}^{m}=(x_{N-m},x_{N-m+1},...,x_{N}) \end{matrix}$$

- We calculate the correlation integral, which measures the spatial correlation between points, by adding the number of pairs of points (*i*, *j*), where$1\leq i\leq N$and$1\leq j\leq N$ in the$m$-dimensional space that are “close” in the sense that the points are within a radius or tolerance$\in$ each other.

$C_{\in,m}=\frac{1}{N_{m}(N_{m}-1)}\sum_{i\neq j} I_{i,j;\in}$ (2)

where$I_{i,j;\in}=\left\{\begin{matrix} 1\mathop{}\limits if\mathop{}\limits\left\|x_{i}^{m}-x_{j}^{m}\right\|\leq\in\\ 0\mathop{}\limits otherwise \end{matrix}\right.$

- Brock, Dechert and Scheinkman (1987) showed that if the time series is I.I.D.

$C_{\in,m}\approx C_{\in,1}$ (3)

where $C_{\in,m}$ is the distributed estimator for dimension $m$ and radius $\in$ is:

$\hat{C}_{\in,m}=\frac{1}{M(M-1)}\sum_{i=1}^{M} \sum_{\begin{matrix} j=1 \\ j\neq i \end{matrix}}^{M} I_{\in}\left(x_{i}^{(m)},x_{j}^{(m)}\right)$ (4)

and $C_{\in,1}$

$\hat{C}_{\in,1}=\frac{1}{T(T-1)}\sum_{i=1}^{T} \sum_{\begin{matrix} j=1 \\ j\neq i \end{matrix}}^{T} I_{\in}\left(x_{i},x_{j}\right)$ (5)

- The BDS statistic for dimension $m$is:

(6)

where $\hat{s}_{m}(\in)$ is the estimator of the asymptotic standard deviation of the numerator. Under fairly moderate conditions the BDS statistic converges to the standard normal distribution.

BDS test is a two-tailed test, we should reject the null hypothesis if the BDS test statistic is greater than or less than the critical values (e.g. if a=0.05, the critical value = ±1.96).

**Unit root testing**

**Zivot-Andrews test**

Zivot-Andrews (1992), following the form of Perron's (1989) models, propose a variation of Perron's (1989) test in which they assume that the exact time of the structural breakpoint is unknown. Considering that the breakpoint is an endogenous phenomenon, Zivot and Andrews propose the following three models:

A. Model with Intercept

(7)

B. Model with Trend

(8)

C. Model with Both Intercept and Trend

(9)

where $DU_{t}$ is a dummy variable for the mean shift and appears in every possible change, whereas $DT_{t}^{*}$ is the corresponding variable for mean shift and trend.

$$DU_{t}\left(\lambda\right)=\left\{\begin{matrix} 1,\mathop{}\limits\mathop{}\limits\mathop{}\limits\alpha\nu\mathop{}\limits t>T\lambda\\ 0\mathop{}\limits\mathop{}\limits\mathop{}\limits\mathop{}\limits otherwise \end{matrix}\right.$$

$$DT_{\tau}^{*}\left(\lambda\right)=\left\{\begin{matrix} t-T\lambda,\mathop{}\limits\mathop{}\limits\mathop{}\limits\alpha\nu\mathop{}\limits t>T\lambda\\ 0\mathop{}\limits\mathop{}\limits\mathop{}\limits\mathop{}\limits otherwise \end{matrix}\right.$$

The above models are based on Zivot-Andrews (1992) models, as a data-dependent algorithm is used as a proxy for Perron to determine the structural points.

The null hypothesis in all three models is:

$H_{0}:\hat{\alpha}=0$ which implies that the series $y_{t}$contains a unit root with a shift that excludes any structural break.

The alternative hypothesis is:

$H_{1}:\hat{\alpha}<0$ which implies that the series $y_{t}$is a trend-stationary process with a one-time break occurring at an unknown point in time.

**2 Nonlinear unit root test**

Nonlinear unit root tests are used to determine whether a time series is stationary when its behavior is nonlinear, meaning it does not have a constant mean, or variation over time. These tests take into account nonlinear patterns using models such as the exponential smooth transition autoregressive model (ESTAR). The univariate model of the nonlinear exponential smooth transition autoregressive process (ESTAR) has the following form:

$y_{t}=\beta y_{t-1}+\gamma y_{t-1}\left[1-\exp\left\{-\vartheta\left(s_{t}-c\right)^{2}\right\}\right]+e_{t}$ (10)

where $y_{t}$ is the series under analysis, $\beta$ and $\gamma$are unknown parameters, $\vartheta\geq0$ is the slope parameter and provides the transition speed to the mean reversal, and$e_{t}\to iid(0,\sigma^{2})$.

**5.3 Kapetanios, Shin, Snell (KSS) Unit Root Test**

Kapetanios et al., (2003) used an ESTAR model with a restructuring by putting$c=0$ and replacing the transient variable$s_{t}$ from a lagged dependent variable$y_{t-d}$ for $d>0$ so that the above equation becomes:

$\Delta y_{t}=\phi y_{t-1}+\gamma y_{t-1}\left[1-\exp\left\{-\vartheta y_{t-d}^{2}\right\}\right]+e_{t}$ (11)

Kapetanios et al., (2003) show by putting$\phi=0$, and $d=1$ in the above equation that the$y_{t}$ follows a unit root procedure and the ESTAR model becomes (see Kapetanios et al., 2003 p. 363).

$\Delta y_{t}=\gamma y_{t-1}\left[1-\exp\left\{-\vartheta y_{t-1}^{2}\right\}\right]+e_{t}$ (12)

where $\gamma$an unknown parameter and the errors are independent and normally distributed$e_{t}\to i.i.d$with zero mean and constant variance $N(o,\sigma^{2})$.

Furthermore, Kapetanios et al., (2003) used the first-order Taylor series in model (12) to obtain the following auxiliary regression:

$\Delta y_{t}=\rho y_{t-1}^{3}+\sum_{i=1}^{k} \lambda\Delta y_{t-i}+e_{t}$ (13)

The two assumptions of equation (13) are written as follows:

$H_{0}:\rho=0$ (unit root or non-stationary).

$H_{1}:\rho<0$ (non-linear stationary ESTAR)

The above hypotheses are tested by the statistic, where$\hat{\rho}$ is the estimate of $\rho$from the auxiliary regression (13).

**5.4 Kruse Unit Root Test**

Kruse, (2011) extends the unit root test of Kapetanios et al., (2003) by allowing the parameter $c\neq0$to be non-zero in the ESTAR model. Kruse (2011) proposes estimating the following model with the assumption $c\neq0$. That is, he examines a nonlinear stationary exponential smooth autoregressive transition (ESTAR) against the null hypothesis of a unit root

$\Delta y_{t}=\phi y_{t-1}\left\{1-\exp\left[-\gamma\left(y_{t-1}-c\right)^{2}\right]\right\}+e_{t}$ (14)

Kruse (2011) using the Taylor approach transforms the above equation as follows:

$\Delta y_{t}=\delta_{1}y_{t-1}^{3}+\delta_{2}y_{t-1}^{2}+\sum_{j=1}^{p} \phi_{j}\Delta y_{t-j}+e_{t}$ (15)

The two assumptions of equation (15) are written as follows:

$H_{0}:\delta_{1}=\delta_{2}=0$ (the series follows a linear unit root process).

$H_{1}:\delta_{1}<0,\delta_{2}\neq0$ (the series follows a nonlinear stationary process of the form ESTAR).

The above hypotheses are tested by the statistics proposed by Abadir and Distaso (2007).

**5.5 Sollis Unit Root Test**

Sollis (2009) developed a nonlinear unit root check for the nonlinear exponential smoothing transitional autoregressive model (ESTAR) that takes into account symmetric or asymmetric reversions. Using the first-order Taylor series in the ESTAR model, we obtain the following auxiliary regression:

$\Delta y_{t}=\phi_{1}y_{t-1}^{4}+\phi_{2}y_{t-1}^{3}+\sum_{i=1}^{k} k_{i}\Delta y_{t-i}+\varepsilon_{t}$ (16)

The null hypothesis in the above function is:

$H_{0}:\phi_{1}=\phi_{2}=0$ (unit root or non-stationary).

The above hypothesis indicates the non-stationarity of the series, and the alternative hypothesis represents the symmetric or asymmetric ESTAR stationarity. In case of rejection of the null hypothesis, to decide whether the series exhibits symmetric or asymmetric ESTAR stationarity, we make the following assumptions:

$H_{0}:\phi_{2}=0$ (symmetrical stationarity ESTAR).

$H_{1}:\phi_{2}\neq0$ (asymmetrical stationarity ESTAR).

The above hypotheses are tested with the *F* statistic and the critical values ​​are listed in Table 1 of the article by Sollis, (2009).

**5.6 Omay, Emirmahmutoglu and Hasanov Unit Root Test**

Omay, Emirmahmutoglu and Hasanov (2018) use the following equation to model the deterministic and stochastic components of an observed time series$y_{t}$.

$y_{t}=\varphi(t)+u_{t}$ (17)

where $\varphi(t)$ is the nonlinear trend determinant and $u_{t}$is the stochastic deviation from the trend. Omay, Emirmahmutoglu and Hasanov consider two specifications of $\varphi(t)$.

The first specification combines the time-dependent (time-varying) nonlinearity of Leybourne, Newbold and Vougas (1998) and the regime-wise nonlinearity of Sollis's (2009) AESTAR model and models a permanent structural change. The disruption is modeled with the logistic transition function following Leybourne, Newbold and Vougas (1998) based on three alternative models:

Model A: $y_{t}=\alpha_{1}+\alpha_{2}S_{t}(\gamma,\tau)+e_{t}$ (18)

Model B: $y_{t}=\alpha_{1}+\beta_{1}t+\alpha_{2}S_{t}(\gamma,\tau)+e_{t}$ (19)

Model C: $y_{t}=\alpha_{1}+\beta_{1}t+\alpha_{2}S_{t}(\gamma,\tau)+\beta_{2}tS_{t}(\gamma,\tau)+e_{t}$ (20)

where *t* = 1,2,....,T; $e_{t}$ is it a zero-means process; and$S_{t}(\gamma,\tau)$ is the logistic smooth transition (LTR) with sample size *T* given by the following equation:

$S_{t}(\gamma,\tau)=\left[1+\exp\left\{-\gamma(t-\tau T\right\}\right]^{-1}$ (21)

The parameters $\gamma$and $\tau$denote the transition speed and location between two regimes respectively.

In model A$y_{t}$ n is stationary around a mean that changes from$\alpha_{1}$ to$\alpha_{1}+\alpha_{2}$. Model B allows for a constant slope term$\beta_{1}$, while the constant term (intercept) changes from$\alpha_{1}$to$\alpha_{1}+\alpha_{2}$. Model C allows for similar changes in the intercept as well as changes in the slope from$\beta_{1}$to $\beta_{1}+\beta_{2}$.

The second specification of the control uses the Fourier series to model the multiple smooth structural changes.

$\phi(t)=\alpha_{0}+\delta t+\sum_{k=1}^{n} \alpha_{k}\sin\left(\frac{2\pi kt}{T}\right)+\sum_{k=1}^{n} b_{k}\cos\left(\frac{2\pi kt}{T}\right)+e_{t}$ $n<\frac{T}{2}$ (22)

where $n<\frac{T}{2}$ represents the number of frequencies *k* is the selected frequency in the approximation process and$\alpha_{i}$ and $b_{i}$ are the measurements for the amplitude and shift of the sinusoidal components of the determinant function.

The Omay, Emirmahmutoglu and Hasanov (2018) test consists of two parts. The first part of the Omay, Emirmahmutoglu and Hasanov (2018) OEHA test is a combination of the logistic smooth transition (LTR) tests and the Sollis (2009) test, so that it takes into account both a single structural change and the size of the imbalance in the adjustment process. The second part of the Omay, Emirmahmutoglu and Hasanov (2018) OEAB test combines the Fourier ADF and Sollis (2009) tests to capture multiple smooth structural changes with an asymmetric ESTAR-like behavior around the nonlinear trend. The second part of the test uses the integral form of the flexible Fourier form-FFF to capture the multiple structural changes.

The Omay, Emirmahmutoglu, and Hasanov (2018) testing procedure begins with estimating models (18), (19), and (20) by taking the residuals from each model. The residuals are then used to test the OEHA and OEAB regression models presented in equations (23), (24), and (25) respectively.

OEHA

$\Delta e_{t}=\delta_{1}e_{t-1}^{3}+\delta_{2}e_{t-1}^{4}+\sum_{j=1}^{k} \delta_{j}\Delta e_{t-1}+u_{t}$ (23)

OEAB

$\Delta y_{t}=\rho_{1}y_{t-1}^{3}+\rho_{2}y_{t-1}^{4}+c_{1}+c_{2}\sin\left(\frac{2\pi kt}{T}\right)+c_{3}\cos\left(\frac{2\pi kt}{T}\right)+\varepsilon_{t}$ (24)

$\Delta y_{t}=\rho_{1}y_{t-1}^{3}+\rho_{2}y_{t-1}^{4}+c_{1}+c_{2}t+c_{3}\sin\left(\frac{2\pi kt}{T}\right)+c_{4}\cos\left(\frac{2\pi kt}{T}\right)+\varepsilon_{t}$ (25)

If the null hypothesis of a linear unit root is rejected against the alternative hypothesis of the OEHA test, this implies that the series is stationary with a structural change with asymmetric ESTAR-like behavior around a nonlinear trend in its structure.

If the null hypothesis of a linear unit root is rejected against the alternative hypothesis of the OEHB test, this implies that the series is stationary around multiple structural changes with asymmetric ESTAR-like behavior around a nonlinear trend in its structure.
